# The health and economic burden of podoconiosis in Ethiopia

**DOI:** 10.1093/trstmh/traa003

**Published:** 2020-02-14

**Authors:** Kebede Deribe, Nebiyu Negussu, Melanie J Newport, Gail Davey, Hugo C Turner

**Affiliations:** 1 Global Health and Infection Department, Brighton and Sussex Medical School, Brighton, BN1 9PX, UK; 2 School of Public Health, College of Health Sciences, Addis Ababa University, P.O.Box 9086, Addis Ababa, Ethiopia; 3 Federal Ministry of Health, P.O.Box 1234, Addis Ababa, Ethiopia; 4 Centre for Tropical Medicine and Global Health, Nuffield Department of Medicine, University of Oxford, OX3 7LG, Oxford, UK; 5 Oxford University Clinical Research Unit, Wellcome Africa Asia Programme, 764 Vo Van Kiet, Quan 5, Ho Chi Minh City, Vietnam

**Keywords:** burden, cost, economic, lymphoedema, podoconiosis

## Abstract

**Background:**

Podoconiosis is one of the leading causes of lymphoedema-related morbidity in low-income settings, but little is known about the scale of its health and economic impact. This information is required to inform control programme planning and policy. In this study, we estimated the health and economic burden of podoconiosis in Ethiopia.

**Methods:**

We developed a model to estimate the health burden attributed to podoconiosis in terms of the number of disability-adjusted life years (DALYs) and the economic burden. We estimated the economic burden by quantifying the treatment and morbidity-management costs incurred by the healthcare system in managing clinical cases, patients' out-of-pocket costs and their productivity costs.

**Results:**

In 2017, there were 1.5 million cases of podoconiosis in Ethiopia, which corresponds to 172 073 DALYs or 182 per 100 000 people. The total economic burden of podoconiosis in Ethiopia is estimated to be US$213.2 million annually and 91.1% of this resulted from productivity costs. The average economic burden per podoconiosis case was US$136.9.

**Conclusions:**

The national cost of podoconiosis is formidable. If control measures are scaled up and the morbidity burden reduced, this will lead to Ethiopia saving millions of dollars. Our estimates provide important benchmark economic costs to programme planners, policymakers and donors for resource allocation and priority setting.

## Introduction

Podoconiosis is a non-infectious geochemical disease that causes massive swelling of the lower leg,^1,2^ with an estimated 4 million cases in 32 countries.^3^ The disease is caused by long-term exposure to soils and thrives in tropical highland areas in genetically susceptible people who do not use footwear. The disease is a disabling neglected tropical disease (NTD) and is associated with profound stigma,^4^ discrimination^5^ and comorbid mental health conditions.^6^ It is the principal cause of lymphoedema in Ethiopia^7,8^; the country bears the highest burden of podoconiosis globally, with an estimated 35 million people at risk and 1.5 million cases across 345 districts.^9,10^ Poor awareness of the condition, weak integration of podoconiosis interventions with primary healthcare delivery, inadequate donor support and resource allocation and scant evidence of the health and economic burden have contributed to the continued burden of podoconiosis in the country.

Podoconiosis is a development challenge in endemic countries. In addition to its health consequences, podoconiosis imposes significant economic burdens on individuals and households through treatment costs and reduced productivity.^11^ A previous study established the association between household food insecurity and presence of podoconiosis.^12^ Podoconiosis is highly prevalent in the central highlands of Ethiopia, which are inhabited by agrarian communities, and so potentially affects the agricultural productivity of the country.^9^

The effectiveness of podoconiosis interventions have been documented. Podoconiosis is preventable through consistent use of footwear and foot hygiene.^13^ The WHO recommends the following basic package of care for patients: treatment for episodes of adenolymphangitis (ADL) and management of lymphoedema to prevent episodes of ADL and progression of disease.^13^ Previous studies have documented the effectiveness of this WHO-recommended hygiene-based management,^14^ which reduces the frequency of episodes of ADL and improves quality of life.^15,16^ Nonetheless, according to data from 2017, in Ethiopia only 12% of endemic districts and 3% of total cases had access to services.^17,18^ To achieve the goal of treatment coverage, it is critical to address the cost barriers to patients.

Despite posing a significant health and economic burden among patients, the overall health and economic burden of podoconiosis in endemic countries is largely unknown, even among high burden countries.^13^ For greater understanding of the podoconiosis burden and to make the case for investment, alternative financing mechanisms and to scale up interventions, it is important that public health policymakers, programme planners and implementers are provided with robust estimates of the health and economic burden of podoconiosis. While previous efforts to estimate the health burden have focused on the number of podoconiosis cases, no studies have estimated its burden in terms of disability-adjusted life years (DALYs).^9,19^ Efforts to estimate the economic burden of podoconiosis have been limited to specific localised endemic areas at the subnational level^11,20^ and national level estimates are not available.

The main objective of this study was, therefore, to estimate the health and economic burden of podoconiosis in Ethiopia.

## Materials and methods

Building on previous methods successfully applied to estimate the health and economic burden of lymphatic filariasis (LF),^21,22^ we developed a model to estimate the health and economic burden of podoconiosis in Ethiopia. Our model incorporated up-to-date data from a variety of sources^21,23–26^ and all costs were adjusted for inflation to 2017 prices.^27,28^

A database was created based on data extracted from numerous online sources.^24,25,29^ These data sources were used in previous analyses.^21,22^. These analyses were conducted using Microsoft Excel (Microsoft, Seattle, USA).

### Epidemiology of podoconiosis in Ethiopia

To estimate the health and economic burden of podoconiosis, it is critical to know the number of cases and the associated disease sequelae. To establish this we used the previously published estimation (developed using geostatistical methods) of 1537 963 podoconiosis cases in Ethiopia in 2017 (uncertainty interval [UI], 290 923–4577 031).^9^ We extracted data from previous studies and found that of those with podoconiosis, 94% may experience ADL episodes,^30–33^ with an average incidence of 5.6 episodes per year (Table 1).^15,30–33^

**Table 1:** Model parameters and the ranges used within the sensitivity analyses

Parameter	Point estimate	Min.	Max.	Sources
Epidemiological model				
Number of cases	1537 963	290 923	4577 031	9
Number treated annually with morbidity-management services	25 000	15 000	50 000	17
Proportion of podoconiosis cases who experience at least one ADL episode	94%	77	98	19–22
Annual incidence of ADL episodes (per those who experience ALD episodes per year)	5.6	5.5	23.3	19–23
Average duration of an ADL episode (d)	4.4	3	6.4	19–23
Productivity costs associated with podoconiosis morbidity				
Reductions in productivity				
During an ADL episode	77.5%	72.5%	84.5%	24
With lymphoedema	45.0%	29.0%	83.0%	11
Economic value of a lost productive day				
GDP per capita of the lowest income quintile	US$0.84	US$0.42	US$1.27	25
Health burden				
DALY disability weights				
ADL disability weight	0.051	0.032	0.074	26
Lymphoedema disability weight	0.109	0.073	0.154	26
Costs related to patients seeking treatment without access to morbidity-management services				
Cost per visit: chronic patients				
Public facility: health system (US$)	0.58	0.47	0.70	27
Public facility: patient (US$)	0.51	0.41	0.61	28–30
Private facility (US$)	1.28	1.02	1.53	28–30
Self-treatment (US$)	0.64	0.51	0.77	28–30
Cost per visit: ADL patients				
Public facility: health system (US$)	0.58	0.47	0.70	27
Public facility: patient (US$)	1.79	1.43	2.15	28–30
Private facility (US$)	3.47	2.77	4.16	28–30
Self-treatment (US$)	0.64	0.51	0.77	28–30
Costs for patients accessing morbidity-management services				
Patients direct treatment cost per year (US$)^*^	22.01	17.61	26.41	31
Patients direct non-medical cost per year (US$)^*^	26.3	21.04	31.56	11
Health system cost per year (US$)^*^	7.91	6.33	9.49	11

Abbreviations: ADL, adenolymphangitis; DALY, disability-adjusted life year; GDP, gross domestic product.

^*^The original data related to a 3-mo period and was adjusted to an annual value.

Costs are in 2017 US$ prices and when necessary are adjusted for inflation.

### Health burden

The health burden of podoconiosis was measured in DALYs with one DALY equating to one healthy year of life lost. A DALYs is a sum of the years of life lost due to premature mortality and the years lost due to disability (YLD) for people with the health condition or its consequences. Due to an absence of data, no excess mortality due to podoconiosis was assumed in the current analysis. DALYs were calculated based on the YLD for two types of morbidities: lymphoedema and ADL episodes. Podoconiosis and LF have similar morbidities in terms of lymphoedema and ADL episodes. Therefore, the disability weightings were based on those used for LF within the Global Burden of Disease (GBD) 2017 Study (Table 1). To account for disability during ADL episodes, we considered an average duration of 4.4 d with a frequency of 5.6 times per annum per patient^15,30–33^ and a disability weight of 0.051 (UI: 0.032–0.074).(34) For lymphoedema we used a disability weight of 0.109 (UI: 0.073–0.154).^34,35^ Based on the methodology employed since the GBD 2010 study, we did not apply a discount rate or age weighting to the DALY estimates.^36,37^ Since lymphoedema and ADL episodes will coexist, we have accounted for the overlap in our estimation of burden by using the multiplicative adjustment method.^35,38^

### Economic burden

The annual economic burden was calculated accounting for the productivity costs associated with podoconiosis morbidity, the costs for patients accessing morbidity-management services and the costs related to patients seeking treatment without access to morbidity-management services. Cost data were standardised to 2017 US$ prices and when necessary adjusted for inflation using gross domestic product (GDP) deflators.^27,28^

### Productivity costs associated with podoconiosis morbidity

The estimated reduction in productivity for lymphoedema and acute episodes associated with podoconiosis was based on previous studies on podoconiosis and LF (Table 1).^39^ When quantifying the total number of days with reduced productivity per year, it was assumed that podoconiosis cases would be potentially economically active for 300 d per year, 8 h per day (Appendix, Table S5). This approach does not differentiate between lost paid or unpaid work (e.g. economic activity can include time spent on household chores or subsistence farming).^21^ Potential double counting of the productive losses from comorbid lymphoedema and ADL episodes was accounted for.

The productivity costs were quantified using the same approach as a recent study on the economic burden of LF.^21^ The human capital approach was used whereby all potential production not performed by a person because of morbidity or early mortality is counted as production loss.^40^ The estimated number of lost productive days were valued based on the GDP per capita of the lowest income quintile (as used by Redekop et al.)^41^ For Ethiopia this was equivalent to US$0.84 per productive day (Table 1).

### Costs relating to the patients accessing morbidity-management services

A proportion of patients access formal morbidity-management services specialised to treat morbidities related to lymphoedema based on WHO-recommended packages of care. Based on reports from implementing partners and health management information data, it is estimated that 25 000 patients are treated annually. The treatment components include cleaning of limbs with diluted antiseptic solutions, soap and water, bandaging, the application of emollient to the skin and provision of shoes for selected patients. Each patient visits a health facility four times over 12 mo to complete their morbidity management.

The direct costs for patients accessing morbidity-management services were stratified into three components (Table 1): patients' direct treatment costs (costs for treatment supplies), patients' direct non-medical costs (including expenditure on travel, lodging and food for patients and accompanying persons) and health system costs. The health system costs were based on the staff time required to provide the services.^11^ The costs were adjusted for inflation to 2017 prices using local inflation rates.

Based on the current available literature, patients would lose 16 d per year accessing the morbidity-management services and a proportion would also be escorted by informal caregivers, who would also incur productivity losses (Appendix Table S5 ). These were valued as productivity costs using the same method as outlined for the productivity costs associated with podoconiosis morbidity.

### Costs related to patients seeking treatment without access to morbidity-management services

**Figure 1:**
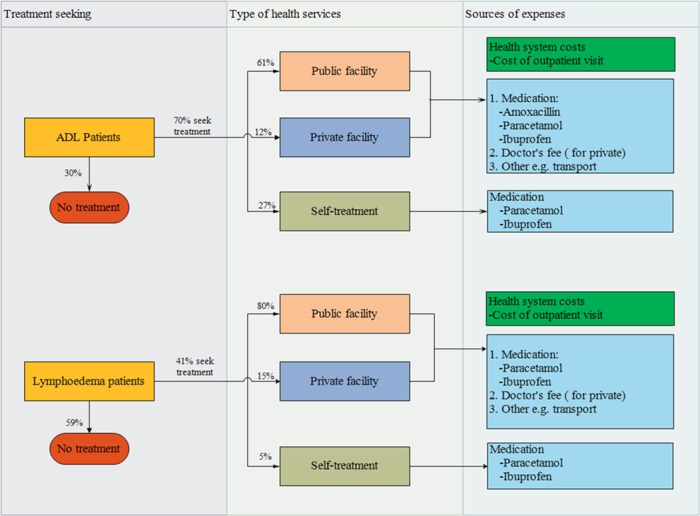
Treatment-seeking behaviour of podoconiosis patients. The sources of the parameters are outlined in Table S5.

Even among patients without access to specialised morbidity-management services, some seek generic care.^32,33^ Among these patients, three treatment-seeking scenarios were assumed: seeking treatment from public health facilities, from private facilities or self-treatment/use of traditional healers.^21^ The proportion of patients seeking care at public and private facilities or opting for self-care are summarised in Figure 1.^23,33^ The costs of utilising services from a health facility include the cost of medication, consultation fees and other costs such as travel and cost to the health system. The costs for these different types of treatment visits were taken from a similar analysis performed for LF^21^ adjusted to 2017 prices (Table 1; US inflation rates were used as the majority of treatment costs related with medications).

It was assumed that medication would include analgesia (ibuprofen), antipyretics (paracetamol) and, in the case of ADL, antibiotics (amoxicillin).^42,43^ Treatment of ADL episodes was assumed to be based on the use of these three medications for 7 d: amoxicillin (500 mg three times a day), paracetamol (500 mg three times a day) and ibuprofen (400 mg twice a day).^44^ Treatment for those with chronic symptoms was assumed to be the same but without amoxicillin (Figure 1). Those who self-treat were assumed to incur lower costs, as they do not require transport, pay consultation fees or receive antibiotics (Table 1, Figure 1).

There is also a cost to the health system associated with patients seeking treatment at public health facilities. As with a similar analysis,^21^ we assumed this to be equivalent to the health system cost associated with an outpatient visit to a rural health facility. We used cost estimates from the WHO-CHOICE database^29^ adjusted to 2017 prices using local inflation rates (Table 1).

### Sensitivity analysis

To test changes in economic burden due to variations in the parameters, we conducted univariate sensitivity analyses. Ranges of parameter values were obtained from a literature search and values from previous LF analyses.^21^ Relevant parameters (such as the disability weights and treatment-seeking behaviour) were grouped together within the sensitivity analyses.

## Results

### Health burden

In 2017, the number of people living with podoconiosis was estimated to be 1537 963 (UI: 290 923–4577 031), with an annual incidence of 8 million ADL episodes among all patients. This corresponds to 172 073 DALYs or 182 per 100 000 population. The majority (97.4%) of these DALYs were due to chronic lymphoedema, while 2.6% were attributable to ADL episodes (Table 2).

**Table 2:** Health burden and lost days in Ethiopia in 2017

Variable	Lymphoedema (hundreds)	ADL episodes (hundreds)	Total (hundreds)
Cases (episodes)	15 379	80 958	
DALYs	1676	44	1720
Productive days lost	2077 237	226 905	2304 142

Abbreviations: ADL, adenolymphangitis; DALYs, disability-adjusted life years.

### Economic burden

We estimated that in 2017 the annual total economic burden due to podoconiosis in Ethiopia was US$213.2 million. The cost related to patients seeking treatment without access to morbidity-management services was US$17.2 million (8.1%), the cost of patients accessing morbidity-management services was US$1.8 million (0.9%) and the productivity cost associated with podoconiosis morbidity was US$194.1 million (91.1%) (Table 3). We also estimated that there were 230.4 million productive days lost per year due to podoconiosis, of which 90.2% were due to chronic illness and 9.8% to acute episodes (Table 2).

**Table 3:** Breakdown of annual economic burden of podoconiosis by disease sequelae

Variable	Lymphoedema (US$ thousands)	ADL episodes (US$ thousands)	Total (US$ thousands)
Productivity costs associated with podoconiosis morbidity	174 972	19 159	194 131 (91.1%)
Costs related to patients seeking treatment without access to morbidity-management services	1012	16 238	17 250 (8.1%)
Costs for patients accessing morbidity-management services	1827	-	1827 (0.9%)
Total	177 811 (83.4%)	35 397 (16.6%)	213 208

Abbreviation: ADL, adenolymphangitis.

Costs are in 2017 US$ prices.

The annual costs related to patients seeking treatment without access to morbidity-management services (consisting of health system costs and out-of-pocket patient costs) totalled US$17.25 million; 94.1% of these costs were due to ADL episodes. By comparison, ADL episodes only accounted for 9.9% of productivity costs associated with podoconiosis morbidity (Table 3).

The average weighted annual economic burden per podoconiosis case was estimated to be US$136.9, the majority of which resulted from lost productivity (US$126.2). On average, the cost related to patients seeking treatment without access to morbidity-management services was US$11.4 per case annually. We estimated that the average annual economic cost of morbidity management and the associated cost per podoconiosis case treated was US$73.1 (Table 4).

**Table 4:** Average economic burden per podoconiosis case

Cost category	Average economic burden per podoconiosis case
Costs related to patients seeking treatment without access to morbidity-management services	US$11.4
Costs for patients accessing morbidity-management services (per case treated)	US$73.1
Productivity costs associated with podoconiosis morbidity	US$126.2
Total (weighted average)	US$136.9

Costs are in 2017 US$ prices.

### Sensitivity analysis

**Figure 2:**
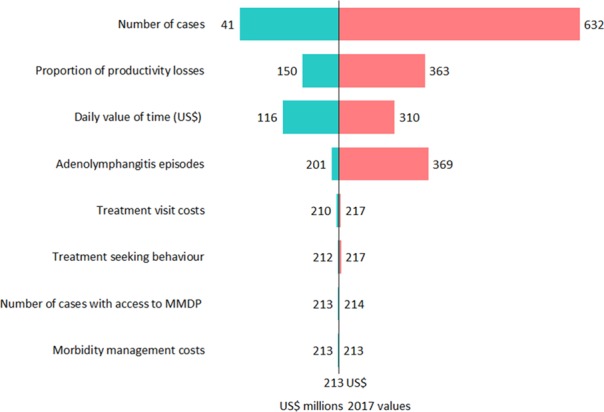
Tornado plot illustrating the impact of univariate sensitivity analysis on the estimated economic burden of podoconiosis. The parameters used for the sensitivity analysis are included in Table 1 and Table S5.

Applying univariate sensitivity analysis, the economic burden was most sensitive to the assumed number of cases. The economic burden was also sensitive to the proportion of productivity losses due to lymphoedema and ADL episodes, incidence of ADL episodes and daily value of time. Parameters reflecting the number of cases with access to morbidity management, treatment-seeking behaviours and treatment costs per visit did not have a notable impact (Figure 2). The total economic burden ranges between US$26.1 million to US$1.6 billion when the parameters are minimised and maximised, respectively.

## Discussion

On the basis of 1.5 million cases of podoconiosis in Ethiopia, we estimated that podoconiosis was responsible for close to 172 000 DALYs annually. The average economic burden per podoconiosis case was US$136.9 and 90% of this resulted from productivity costs. The total economic burden of podoconiosis in Ethiopia is estimated to be close to US$213.2 million annually. The assumed number of podoconiosis cases was based on a nationwide mapping and robust modelling approach.^9,10^ This provides a strong foundation for the estimation of the economic burden of podoconiosis in Ethiopia. Our findings demonstrate that podoconiosis is an important NTD causing significant health and economic burdens. They reaffirm that podoconiosis is not only an important public health problem but also a formidable developmental challenge for the country.

The average annual cost per patient (US$136.9) is comparable with figures estimated for LF.^21^ In the Ethiopian context, the figure is significant in a country where more than a quarter of the population live below the international poverty line (US$1.9 per capita per day)^45^ and where the poverty level is higher in rural areas,^45^ where podoconiosis is most prevalent. The number of productive days lost due to podoconiosis and the incidence of ADL per annum are considerable. These all emphasise the need to scale up morbidity-management services for affected individuals. Previous studies have demonstrated that lymphoedema-related morbidity management significantly reduces the frequency of acute episodes,^15^ is likely to be cost-effective and generate economic benefits.^22,46,47^. Our estimates differ from a previous estimate of the cost per patient for podoconiosis; this difference is due to our approach in calculating direct and indirect costs.^11^

Our study also provides the first nationwide burden estimates for podoconiosis. The burden of podoconiosis is possibly under-recognised due to misclassification as other diseases. The national burden of podoconiosis as expressed in DALYs (172 073) in 2017 is higher than that of trachoma (15 672), onchocerciasis (40 558) and leishmaniasis (45 900), although less than schistosomiasis (178 312) and malaria (201 482).^35^ In Ethiopia in 2017, all NTDs considered in the GBD estimation caused 547 599 DALYs.^35^ If podoconiosis was included in the GBD 2017 estimation it would have accounted for 24% of the total NTD DALYs and 0.5% of the total all-cause DALYs in Ethiopia.^35^

This is the first estimate to quantify the health and economic burden of podoconiosis in Ethiopia. The epidemiology of podoconiosis and the number of podoconiosis cases estimated is based on nationwide mapping and a robust modelling approach, both of which provide a strong foundation for the estimation of the economic burden. We used a study conducted among podoconiosis patients to estimate economic costs and productivity loss.^11^

The economic burden of podoconiosis was US$213.2 million annually, based on our sensitivity analysis ranging from US$41 million to US$632 million. The variation was largely driven by the estimated number of podoconiosis cases. Although robust methods were applied in modelling the estimation of cases, there were wide uncertainty intervals in the estimates due to factors potentially unaccounted for in the models.^9^

### Limitations

There are some limitations to our estimation. First, in estimating the health burden of podoconiosis, we only included ADL and lymphoedema and there are other possible sequelae. These include permanent joint fixation and comorbid depression,^6^ but these are not included because of paucity of data. One study found that including mental health sequalae in LF DALY burden estimates resulted in a near doubling of its estimated DALY burden.^48^ In addition, we do not have data on long-term disease outcomes, including excess mortality due to the disease. This emphasises the need for data on other sequelae and long-term outcomes of podoconiosis.

Second, estimates of the proportion of patients seeking care at public and private facilities or opting for self-care are dependent on a single study for ADL^33^ and are based on the national household survey.^23^ Health service utilisation was also estimated based on a study conducted among the general population.^49^ We believe these estimates are reasonably close to the true figure, but it is important that podoconiosis-specific data are generated to improve future estimations of the economic burden.

Third, for determining the productivity costs, we only calculated productivity losses experienced by the patients (with the exception of the caregivers assisting with morbidity management). Nonetheless, it is likely that many economically productive family members miss work, particularly during ADL episodes, to care for patients. Therefore, we recommend a comprehensive community-based survey in a podoconiosis-endemic area to estimate the aforementioned costs and the number of productive days lost by caregivers and family members. Mental health conditions including depression are common among caregivers as well as patients, but we have not estimated the potential economic burden associated with higher prevalence of depression among caregivers.^6^

In addition, accurately valuing productivity losses for individuals with podoconiosis is difficult and the correct methodology is debatable.^21^ In order to be conservative, the GDP per capita of the lowest income quintile was used in this study (Table 1). The human capital approach was used to estimate the productivity costs. However, it should be noted that the friction cost approach (which assumes the employer's perspective for valuing lost productivity),^50^ typically results in lower estimates of productivity costs. There is continued debate within the field regarding which approach is most appropriate.^40^ However, in the context of podoconiosis, the friction cost approach is difficult to apply, as the majority of those affected are not in formal employment. It was assumed that otherwise podoconiosis cases would have been potentially economically active for 300 d per year, considering both paid and unpaid work.^21^ We did not explicitly value lost leisure time. However, it should be noted that distinguishing between the lost unpaid work and leisure time can be challenging.^40^ Furthermore, although it was also not possible to present the results disaggregated by gender, it is probable that women and men face a different economic burden associated with podoconiosis.

Finally, we estimated the economic burden of podoconiosis at national level and we did not show inter-regional variation in the economic burden of podoconiosis because of the lack of region-specific cost data. Therefore, we recommend future studies to address this. Based on the available data, we only performed a univariate sensitivity analysis. However, as more data become available, future studies should consider a probabilistic sensitivity analysis.

To fully understand the cost and economic aspect of podoconiosis interventions, the following studies are recommended. First, most of our costs are dependent on international databases^23–25,29^ and other diseases such as LF,^21,22^ therefore it is important to conduct studies which collect either cross-sectional or longitudinal data on costs related to podoconiosis. It will be important that such studies collect more detailed data on health-seeking behaviours as well as further economic and health burden data on individuals who are self-treating.^51^ Second, our analysis focused only on Ethiopia, and it is important in future to estimate the health and economic burden of podoconiosis in multi county settings (if not at the global scale) as data on the number of cases become available. Third, cost-effectiveness studies on hygiene-based treatment and other preventive interventions are critical.^52^ Previous studies have demonstrated the effectiveness of the morbidity-management intervention,^15,16^ but the cost-effectiveness of the intervention has not yet been evaluated.

### Conclusions

Our estimate of the health and economic burden of podoconiosis suggests that considerable health burdens and economic losses are borne by Ethiopia. Our findings imply that reducing the podoconiosis burden would contribute to poverty reduction in the country. The Ministry of Health should scale up podoconiosis morbidity-management interventions within reasonable access to those living with podoconiosis. Our estimates provide important benchmark economic costs for programme planners, policymakers and donors for resource allocation and priority setting.

## Supplementary Material

Supporting_Information_11_10_2019_traa003
