# Supplementary material for: The health and economic burden of podoconiosis in Ethiopia
Source: Trans R Soc Trop Med Hyg. 2020 Feb 14;114(4):284–92. doi: 10.1093/trstmh/traa003 (PMC7139123; doi:10.1093/trstmh/traa003)
Supplement: Supporting_Information_11_10_2019_traa003 [file supporting_information_11_10_2019_traa003.docx]

**SUPPLEMENTARY APPENDIX**

**The Health and Economic Burden of Podoconiosis in Ethiopia**

Kebede Deribe PhD^1,2*^, Nebiyu Negussu MPH^3^, Melanie J. Newport PhD^1^, Gail Davey MD^1,2^, Hugo C. Turner PhD^4,5^

1. Wellcome Trust Brighton and Sussex Centre for Global Health Research, Brighton and Sussex Medical School, Brighton, BN1 9PX, UK
2. School of Public Health, College of Health Sciences, Addis Ababa University, Addis Ababa, Ethiopia
3. Federal Ministry of Health, Addis Ababa, Ethiopia
4. Centre for Tropical Medicine and Global Health, Nuffield Department of Medicine, University of Oxford, Oxford, United Kingdom
5. Oxford University Clinical Research Unit, Wellcome Africa Asia Programme, Ho Chi Minh City, Vietnam.

**Table of Contents**

[Table S1. Health burden and lost days in Ethiopia in 2017 3](#_Toc15643025)

[Table S2: Costs related to patients seeking treatment without access to morbidity management services. 3](#_Toc15643026)

[Table S3: Costs for patients accessing morbidity management services (per case treated) 3](#_Toc15643027)

[Table S4: Total economic burden of podoconiosis 4](#_Toc15643028)

[Table S5: Parameters used in the analysis 5](#_Toc15643029)

| **Table S1. Health burden and lost days in Ethiopia in 2017** | | | |
| --- | --- | --- | --- |
| **Variables** | **Lymphoedema** | **ADL episodes** | **Total** |
| DALYs | 167638 | 4435 | **172073** |
| Days lost | 207723682 | 22,690,525 | **230414206** |
| *Costs are in 2017 US$ prices* | | | |

| **Table S2: Costs related to patients seeking treatment without access to morbidity management services.** | | | |
| --- | --- | --- | --- |
| **Cost category** | **Lymphoedema US$** | **Adenolymphangitis episode US$** | **Total US$** |
| Treatment cost - patient | 13,405,179 | 583,040 | 13,988,219 |
| Treatment cost - health system | 2,833,147 | 428,805 | 3,261,952 |
| **Total treatment cost per year** | **16,238,326** | **1,011,845** | **17,250,171** |
| *Costs are in 2017 US$ prices* | | | |

| **Table S3: Costs for patients accessing morbidity management services (per case treated)** | | |
| --- | --- | --- |
| **Cost category** | **Total US$** | **Cost per treated case US$** |
| Patient - direct treatment cost | 550,250 | 22 |
| Patient - direct non-medical cost | 657,500 | 26.3 |
| Patient - indirect cost | 337,744 | 13.5 |
| Caregiver - indirect cost | 83,338 | 3.3 |
| Health system cost | 197,724 | 7.9 |
| **Total Morbidity management (cost per year)** | 1,826,556 | 73.1 |
| *Costs are in 2017 US$ prices* | | |

| **Table S4: Total economic burden of podoconiosis** | | |
| --- | --- | --- |
| Cost category | **Total US$** | Percent |
| Productivity costs associated with podoconiosis morbidity | 194,131,457 | 91.1% |
| Total costs related to patients seeking treatment without access to morbidity management services. | 17,250,170 | 8.1% |
| Total costs for patients accessing morbidity management services | 1,826,556 | 0.9% |
| **Total** | **213,208,183** |  |
| *Costs are in 2017 US$ prices* | | |

| **Table S5: Parameters used in the analysis** | | | | |
| --- | --- | --- | --- | --- |
| **PARAMETERS** | **BASELINE** | **LOW** | **HIGH** | **REFERENCES** |
| **Epidemiological model** |  |  |  |  |
| Number of cases | 1,537,963 | 290,923 | 4,577,031 | ^1^ |
| Number with morbidity management | 25000 | 15000 | 50000 | ^2^ |
| **PRODUCTIVITY** |  |  |  |  |
| Productive days per year | 300 | 260 | 365 | ^3^ |
| Daily value of time ($) - 2017 prices | 0.84 | 0.42 | 1.27 | ^3^ |
| **Productivity loss due to disease** |  |  |  |  |
| During an Adenolymphangitis (ADL) episode | 77.50% | 72.50% | 84.50% | ^4^ |
| With lymphoedema | 45.00% | 29.00% | 83.00% | ^5^ |
| **ADENOLYMPHANGITIS EPISODES** |  |  |  |  |
| Proportion of podoconiosis cases who experience at least one ADL episode | 94% | 77% | 98% | ^6-9^ |
| Annual incidence of ADL episodes (among people with podoconiosis per year) | 5.6 | 5.5 | 23.3 | ^6-10^ |
| Average duration of an ADL episode (days) | 4.4 | 3 | 6.4 | ^6-10^ |
| **Costs related to patients seeking treatment without access to morbidity management services** | | | | |
| **Chronic patients: Lymphoedema** |  |  |  |  |
| Treatment seeking (lymphoedema) | 0.41 | 0.3 | 1 | ^11^ |
| Frequency of Seeking Treatment | 1.50 | 0.5 | 3 | ^11,12^ |
| Public facility (%) | 0.80 |  |  | ^11^ |
| Private facility (%) | 0.15 |  |  | ^11^ |
| Self-treatment (%) | 0.05 |  |  | ^11^ |
| **Cost per visit - chronic** |  |  |  |  |
| Cost per visit chronic- Public facility: health system (US$) | 0.58 | 0.47 | 0.70 | ^13^ |
| Cost per visit chronic - Public facility US$ | 0.51 | 0.41 | 0.61 | ^3,14,15^ |
| Cost per visit chronic - Private facility US$ | 1.28 | 1.02 | 1.53 | ^3,14,15^ |
| Cost per visit chronic - Self-treatment US$ | 0.64 | 0.51 | 0.77 | ^3,14,15^ |
| **ADL episodes** |  |  |  |  |
| Treatment seeking ADL episodes | 0.70 | 0.5 | 1 | ^9^ |
| Public facility (%) | 0.61 |  |  | ^9^ |
| Private facility (%) | 0.12 |  |  | ^9^ |
| Self-treatment (%) | 0.27 |  |  | ^9^ |
| **Cost per visit -** ADL episodes |  |  |  |  |
| Cost per visit ADL- Public facility: health system (US$) | 0.58 | 0.47 | 0.70 | ^13^ |
| Cost per visit ADL - Public facility US$ | 1.79 | 1.43 | 2.15 | ^3,14,15^ |
| Cost per visit ADL - Private facility US$ | 3.47 | 2.77 | 4.16 | ^3,14,15^ |
| Cost per visit ADL - Self-treatment US$ | 0.64 | 0.51 | 0.77 | ^3,14,15^ |
| **COSTS FOR PATIENTS ACCESSING MORBIDITY MANAGEMENT SERVICES** | | | | |
| Patient - direct treatment cost per year US$ | 22.01 | 17.61 | 26.41 | ^16^ |
| Patient - direct non-medical cost per year US$ | 26.3 | 21.04 | 31.56 | ^5^ |
| Patient - days lost per year | 16 |  |  | ^5^ |
| Caregivers - days lost per year | 28 |  |  | ^5^ |
| Proportion patients escorted | 14.10% |  |  | ^5^ |
| Health system costs per year US$ | 7.91 | 6.33 | 9.49 | ^5^ |
| **DALY DISABILITY WEIGHTS** |  |  |  |  |
| ADL disability weight | 0.051 | 0.032 | 0.074 | ^17^ |
| Lymphoedema disability weight | 0.109 | 0.073 | 0.154 | ^17^ |
| *Costs are in 2017 US$ prices* | | | | |

**References**

1. Deribe K, Cano J, Giorgi E, et al. Estimating the number of cases of podoconiosis in Ethiopia using geostatistical methods. . *Wellcome Open Res* 2017; **2**(78).

2. Deribe K, Kebede B, Mengistu B, et al. Podoconiosis in Ethiopia: From Neglect to Priority Public Health Problem. *Ethiop Med J* 2017; **55**((Suppl 1)): 65-74.

3. Mathew C, Bettis A, Chu B, et al. The health and economic burden of lymphatic filariasis prior to mass drug administration programmes. *Clin Infect Dis ( In press)* 2019.

4. Lenk EJ, Redekop WK, Luyendijk M, Rijnsburger AJ, Severens JL. Productivity Loss Related to Neglected Tropical Diseases Eligible for Preventive Chemotherapy: A Systematic Literature Review. *PLoS Neglected Tropical Diseases* 2016; **10**(2): e0004397.

5. Tekola F, HaileMariam D, Davey G. Economic costs of endemic non-filarial elephantiasis in Wolaita Zone, Ethiopia. *Tropical Medicine & International Health* 2006; **11**(7): 1136-44.

6. Alemu G, Tekola Ayele F, Daniel T, Ahrens C, Davey G. Burden of podoconiosis in poor rural communities in Gulliso woreda, West Ethiopia. *PLoS Negl Trop Dis* 2011; **5**(6): e1184.

7. Bekele K, Deribe K, Amberbir T, Tadele G, Davey G, Samuel A. Burden assessment of podoconiosis in Wayu Tuka woreda, east Wollega zone, western Ethiopia: a community-based cross-sectional study. *BMJ Open* 2016; **6**(9): e012308.

8. Molla YB, Tomczyk S, Amberbir T, Tamiru A, Davey G. Podoconiosis in East and West Gojam zones, Northern Ethiopia. *PLoS Negl Trop Dis* 2012; **6**(7): e1744.

9. Tekola Ayele F, Alemu G, Davey G, Ahrens C. Community-based survey of podoconiosis in Bedele Zuria woreda, southwest Ethiopia. *Int Health* 2013; **5**(2): 119-25.

10. Negussie H, Molla M, Ngari M, et al. Lymphoedema management to prevent acute dermatolymphangioadenitis in podoconiosis in northern Ethiopia (GoLBeT): a pragmatic randomised controlled trial. . *Lancet Glob Health* 2018; **6**(7): e795-e803.

11. Federal Democratic Republic of Ethiopia Ministry of Health. Ethiopian Health Accounts Household Health Service Utilization and Expenditure Survey 2015/2016. Addis Ababa, Ethiopia; 2017.

12. Bazie GW, Adimassie MT. Modern health services utilization and associated factors in North East Ethiopia. *PLoS One* 2017; **12**(9): e0185381.

13. World Health Organization. Cost effectiveness and strategic planning (WHO-CHOICE): Health service delivery costs. <http://www.who.int/choice/cost-effectiveness/inputs/health_service/en/>. Accessed on 25 June 2019. .

14. Management Sciences for Health. The International Medical Products Price Guide. Avaiable at <http://mshpriceguide.org/en/home/>. (Accessed: 20 March 2019).

15. Health Action International, World Health Organisation. Database of medicine prices, availability, affordability and price components. Available at : <http://www.haiweb.org/MedPriceDatabase/>. Accessed on 28 March 2019. .

16. Tamiru A, Tsegay G, Wubie M, Gedefaw M, Tomczyk S, Tekola-Ayele F. Podoconiosis patients' willingness to pay for treatment services in Northwest Ethiopia: potential for cost recovery. *BMC Public Health* 2014; **14**: 259.

17. GBD 2017 DALYs and HALE Collaborators. Global, regional, and national disability-adjusted life-years (DALYs) for 359 diseases and injuries and healthy life expectancy (HALE) for 195 countries and territories, 1990-2017: a systematic analysis for the Global Burden of Disease Study 2017. *Lancet* 2018; **392**(10159): 1859-922.
